# Detecting the presence-absence of bluefin tuna by automated analysis of medium-range sonars on fishing vessels

**DOI:** 10.1371/journal.pone.0171382

**Published:** 2017-02-02

**Authors:** Jon Uranga, Haritz Arrizabalaga, Guillermo Boyra, Maria Carmen Hernandez, Nicolas Goñi, Igor Arregui, Jose A. Fernandes, Yosu Yurramendi, Josu Santiago

**Affiliations:** 1 AZTI-Tecnalia, Marine Research Division, Pasaia, Spain; 2 Computer Science and Artificial Intelligence Department, University of the Basque Country, Donostia, Spain; 3 Centre for the Research and Technology of Agro-Environmental and Biological Sciences, University of Trás-os-Montes and Alto Douro, Vila Real, Portugal; 4 Plymouth Marine Laboratory, Plymouth, United Kingdom; Technical University of Denmark, DENMARK

## Abstract

This study presents a methodology for the automated analysis of commercial medium-range sonar signals for detecting presence/absence of bluefin tuna (*Tunnus thynnus*) in the Bay of Biscay. The approach uses image processing techniques to analyze sonar screenshots. For each sonar image we extracted measurable regions and analyzed their characteristics. Scientific data was used to classify each region into a class (“tuna” or “no-tuna”) and build a dataset to train and evaluate classification models by using supervised learning. The methodology performed well when validated with commercial sonar screenshots, and has the potential to automatically analyze high volumes of data at a low cost. This represents a first milestone towards the development of acoustic, fishery-independent indices of abundance for bluefin tuna in the Bay of Biscay. Future research lines and additional alternatives to inform stock assessments are also discussed.

## Introduction

The Atlantic bluefin tuna (*Thunnus thynnus*) is an emblematic species exploited for several centuries that has supported economically important industrial fisheries [1]. The International Commission for the Conservation of Atlantic Tunas (ICCAT) manages two Atlantic bluefin tuna stocks, the western stock that spawns in the Gulf of Mexico, and the eastern stock that spawns in the Mediterranean. Both stocks have been overfished in recent decades [2] and currently they are under recovery plans. Furthermore, the scientific community has warned about the large uncertainty surrounding the eastern stock status [3], which is being addressed with a set of research programs under the Atlantic-wide Research Programme for bluefin Tuna (GBYP) promoted by ICCAT. In order to be able to quantify the effects of the implemented recovery plan, it is of outmost importance to be able to monitor changes in abundance and stock status through accurate indicators.

Fisheries independent scientific surveys are used to monitor the stock abundance of many groundfish and small pelagics [4]. Absolute and relative stock abundance estimates are useful to inform management of exploited fish stocks. Many of the uncertainties associated with our ability to estimate fish stock abundances can be linked directly to limitations in the spatial coverage of our sampling systems [5]. For example, in the case of scientific acoustic surveys, highly precise narrow vertical beam acoustic equipment might fail to detect aggregations if these are sparsely distributed or if fish are aggregated in the unsampled surface. In such situations, the use of commercial fishing vessels and their acoustic equipment allows for substantial increase in the spatial coverage. In fact, major progress has been made in the use of this information as the basis for stock assessment [6, 7, 8, 9], as well as to analyze fish behavior [10], vessel avoidance [11] and fish distribution [12].

In tuna stock assessments, time series of standardized catch per unit effort (CPUE) indices are used as proxies for relative abundance. However, these series, based on fishery data, have known analytical challenges, such as lack of scientific design, correlated observations, non-random sampling or variable catchability [13], and do not necessarily reflect trends in population abundance. In the case of bluefin tuna, the drastic reduction in fishing opportunities as part of the recovery plan has affected the CPUE indices, and the Standing Committee on Research and Statistics (SCRS) of ICCAT has recommended urgently developing fisheries independent indices of abundance [14].

There are very few fishery-independent surveys for tuna, and other highly mobile species with wide distributional ranges, because the cost associated with research vessels covering the whole distribution area is prohibitive. Moreover, it is not possible to account for the uncertainty associated with this type of surveying (e.g. double counting). Therefore, some fishery independent surveys for tuna have focused on early life stages (larvae) or spawners whose distributional range is much more concise and spatially limited to spawning areas [15]. When the focus has been on juveniles and adults (with high migration capabilities) airplanes have been used instead of research vessels to provide broad distribution coverage in reasonable timeframes and with reasonable costs [16, 17], estimating the approximate horizontal shape of the visible portion of schools [18]. Some sonar and echosounder-based acoustic surveys have also been implemented to monitor southern bluefin tuna recruitment [19], together with trolling transects surveys [20].

The standardized CPUE of the Bay of Biscay baitboat fleet is used as the only abundance index for the juvenile fraction of the entire eastern stock [21, 22]. Catchability by baitboats can be affected by several factors including food availability, feeding behavior and stomach repletion [23, 24]. These variables are difficult to incorporate during the CPUE standardization process. Consequently, inter-annual variability could induce bias in the abundance indices (e.g. a large tuna biomass could yield a low baitboat CPUE if plenty of food is available in the environment and tunas are not attracted by the bait). However, Bay of Biscay baitboats use Omni mode Medium Range Sonars (MRS) to search for tuna, and omni directional sonars have proven to be useful tools for characterizing large pelagic schools [25, 26]. Thus, the information obtained by these sonars could provide data about the number and size of tuna schools in the search area, independent of food availability and feeding behavior. These sonars are analog and non-scientific, used only for display, and all the information collected is lost as soon as it disappears from the screen. Thus, our approach is to record sonar screen shots in a large number of fishing vessels during the tuna fishing campaigns and design an automated methodology for analyzing these images as a way to utilize the data currently wasted. The automated processing of images has been proven to be useful in biological studies and it is a fast-evolving area of research [27, 28, 29].

In summary, the estimation of bluefin tuna abundance in the Bay of Biscay using fishery independent methods remains challenging, but new technologies, datasets and approaches provide new opportunities to address the challenge. The main objective of this study is to develop an automated image analysis procedure for detecting presence-absence of bluefin tuna in commercial sonar images, plus a validation of the procedure based on data mining. The utility of the procedure to track abundance of juvenile bluefin tuna in the Bay of Biscay is also discussed. This constitutes a first milestone towards the longer-term objective of developing new fishery independent indices of abundance for Atlantic bluefin tuna based on acoustics.

## Materials and methods

The research presented in this manuscript involved no endangered or protected species. No experimentation with animals was performed and no specific field permits were required as the scientific observations were conducted on commercial fishing activities regulated by the International Commission for the Conservation of Atlantic Tunas (ICCAT). No other ethical issues applied to the present research project.

**Fig 1:**
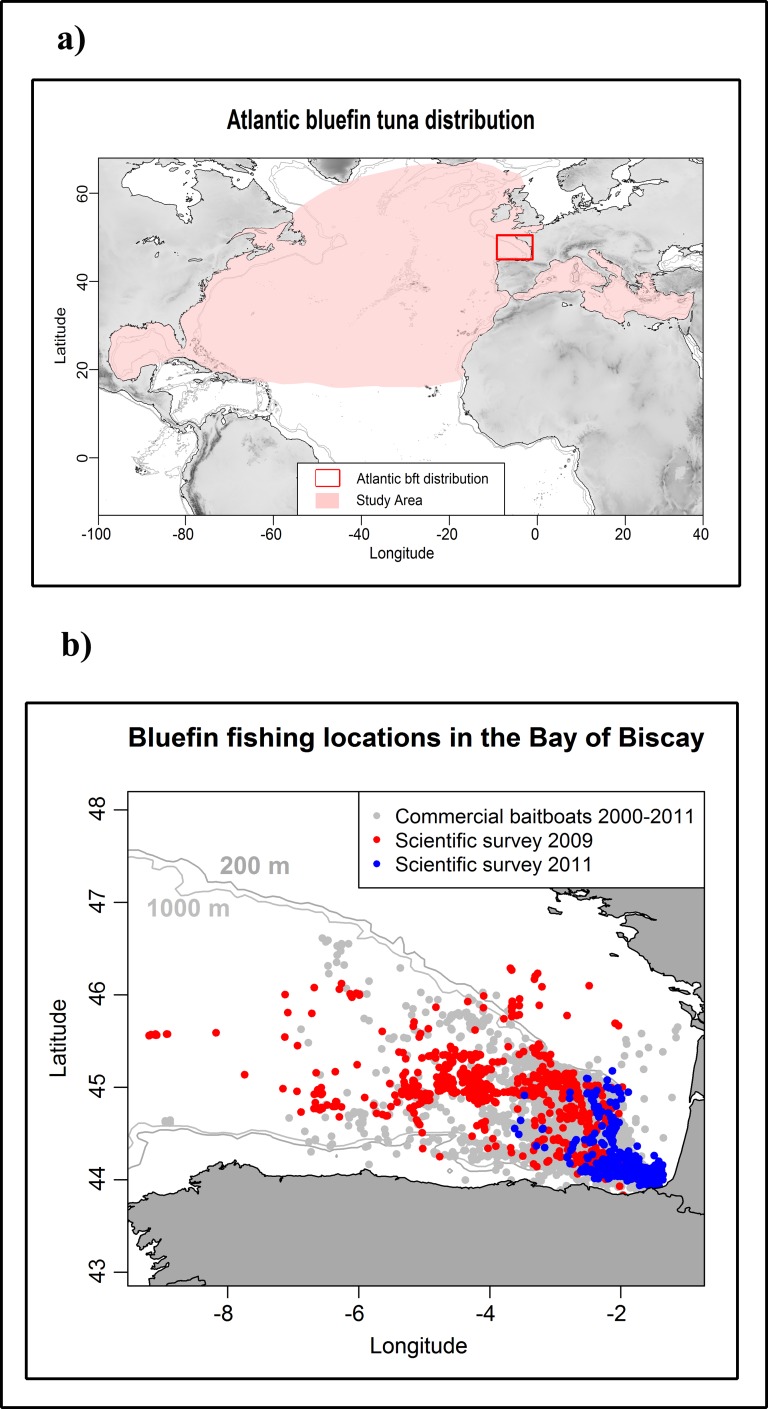
The study area. A) Atlantic bluefin tuna distribution based on ICCAT catch data for the period 2000–2013 [14]. B) The study area, bluefin tuna fishing locations based on logbook data [22] and scientific surveys conducted in 2009 and 2011.

The study area is delimited by the activity of the baitboat fleet in the southeast corner of the Bay of Biscay, between 43–47°N and 2–6°W, from June to October (Fig 1). The Bay of Biscay represents a relatively small fraction of the total bluefin tuna habitat in the Atlantic [30]. However, it is the most important known feeding area for juveniles during their feeding migration to the Northeast Atlantic around summer [31].

Pole and line fishing with live bait is the traditional fishing technique used by the Basque fleet fishing for bluefin tuna in the Bay of Biscay since the early 1950s. Live bait (mainly small horse mackerel, sardine, mackerel and anchovy) is caught with a small purse seine and kept in water tanks onboard. Tuna schools can be spotted visually at large distances and then detected acoustically by sonar, once the school is within the detection range of the sonars. When the boat is close to the tuna school, live bait is thrown into the water to keep the tuna next to the boat, while the boat sprays water so that it is not seen by the tuna. At this point, baited hooks are used to catch the tuna.

In this study we created a reference dataset of sonar images with known categories (“tuna” or “no tuna”, based on tuna presence and absence data observed by scientists) to validate an image analysis and classification procedure. This dataset was used to test the methodology developed in this study which consists of several steps: 1) Image acquisition and categorization based on scientific data, 2) Features extraction, 3) Training dataset elaboration and 4) Model training and evaluation.

### Image acquisition and categorization based on scientific data

The images processed in this study were obtained from the commercial sonar MAQ 90 kHz. This omni-directional MRS is used by the majority of the Bay of Biscay baitboat fleet. The searching range of the sonar varies with sea conditions and skipper preferences but, in general, range settings of 100–300 m are used when searching for tuna, with a slight tilt of minus 5–7° off the horizontal and narrow vertical and horizontal beam widths (5°).

**Fig 2:**
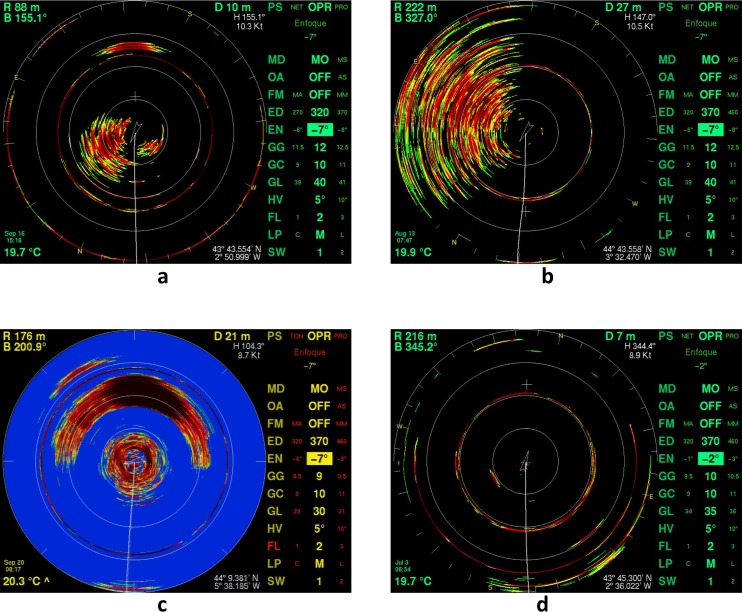
Main types of images recorded. Typical cases of echograms: tuna, black background (a); no tuna, noise (b); tuna, blue background (c) and no tuna (d).

The screen dumps were acquired using an image acquisition device composed of 400MHz video splitter, an external VGA Capture Device and a laptop with a script for continuous data acquisition. The images selected for this study correspond to six different trips from two scientific tuna surveys conducted in summer 2009 and 2011. The scientific surveys were conducted using a baitboat that behaved similar to the rest of the commercial baitboat fleet. Thus, the area searched during the scientific surveys significantly overlapped the fishing area used by the commercial fleet (Fig 1). The main activities conducted by the scientists during the surveys were characterization of the vessel activities, recording of MAQ sonar screenshots and SIMRAD EK 60 signal, tuna tagging and biological sampling (length measurements as well as collection of genetic tissue). The presence of bluefin tuna in the sonar was validated when bluefin tuna was the only specie caught during fishing operations. Presence of bluefin tuna was annotated in the scientific logbooks, and this information was used to classify the images under “tuna” and “no tuna” categories. For this study, the reference dataset was built by selecting a balanced set of images, with 1397 images of bluefin tuna presence and 1398 images of bluefin tuna absence. Bluefin tuna absence was defined as lack of tuna echo in the image and lack of tuna catch. With the aim to include the main types of images recorded, the reference dataset included images with different background colors as well as images with and without surface noise (Fig 2).

### Features extraction

The image processing application was developed with a Java software and it consisted of three steps: pre-processing, segmentation and extraction of characteristics.

#### Pre-processing

**Fig 3:**
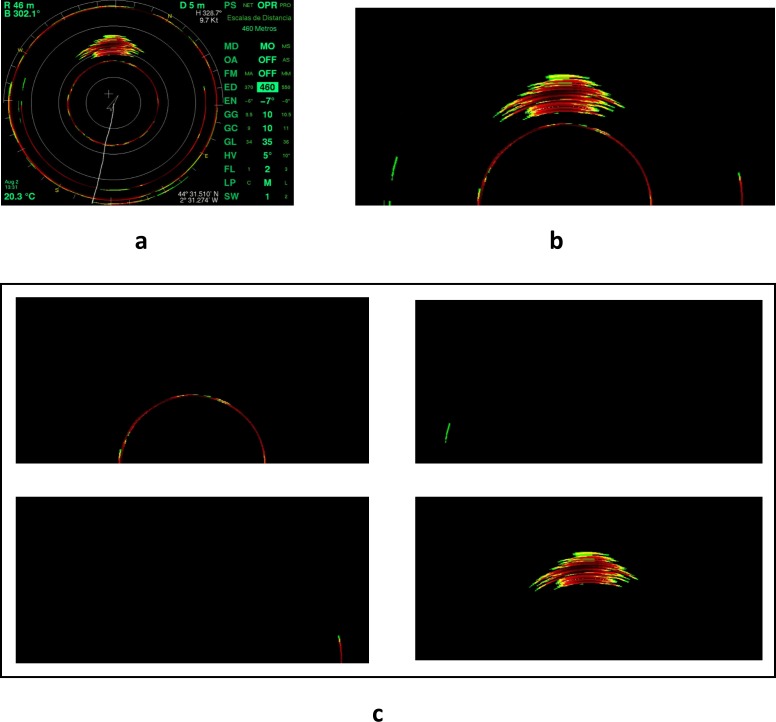
Image pre-processing phase. Sequential steps of the features extraction procedure: (a) original image, (b) image pre-processing, and (c) segmentation of the operative part of the echogram into “blobs”.

The pre-processing phase removed the non-relevant parts of the sonar screen image. The screen of the MAQ sonar has two main regions, the echogram display circle and the menu panel (Fig 2). The menu panel provides user information on the operation and system control settings whereas the echogram represents the acoustic data. During the pre-processing we divided the sonar screen into these two basic regions and then focused on the echogram. In the echogram, we worked with the upper half of the circle, as the tuna schools are not clearly detected in the lower half due to the vessel’s wake. Furthermore, the schools were observed to appear first in the upper part of the echogram because the vessels move faster than the fish. The sonar display was set up in such a way that the forward observations were located at the top of the screen. Additionally, the echogram was cleaned of noise and sonar display lines and marks, such as cursor crosses, vessel tracks or range circumferences were removed from the echogram (Fig 3).

#### Segmentation

In the segmentation phase, the selected part of the echogram was partitioned into sub-images or blobs. First, the zero-valued (i.e., black) pixels were considered background and removed; whereas the non-zero (i.e., colored) pixels were grouped, using the 8 adjacency rule, into blobs. Then, in order to reduce the size of the training dataset, the blobs containing less than 100 pixels were removed. We believe that this decision is conservative since the smallest tuna school observed by expert judgement in the reference dataset contained 415 pixels, and so it does not restrict the utility of the classification algorithm developed.

#### Extraction of characteristics

The remaining blobs were considered tuna candidates and were subject to a characteristics extraction process. For each one, 20 morphologic characteristics were measured related to area, perimeter, position, smallest rectangle containing the blob, best ellipse fitting the blob, aspect ratio, circularity, solidity, greatest distance between any pair of pixels of the blob (known as Feret or Feret’s diameter), the projections of Feret's diameter on the axes, the angle of Feret's diameter with respect to the horizontal axis and the minimum value of the Feret's diameter. Finally, the blobs were labeled with two possible categories: “tuna” and “no-tuna”, according to scientific observations.

### Training dataset elaboration

Based on the reference images, a training dataset of blobs was created to train automatic classification programs and to test their efficiency before they were used to classify new unsupervised images (e.g. those collected onboard commercial fishing vessels without an observer onboard). The training dataset included the categories “tuna” (presence) and “no-tuna” (absence), and is available as S1 Dataset.

From the 1397 presence and 1398 absence images in the reference images, after the features extraction, we obtained 22501 blobs for constructing the training dataset: 1497 were positive examples (presence) and 21004 were negative examples (absence), as shown in Table 1. The resulting ratio between positive/negative instances was 1/14.03, which shows that we had an unbalanced training dataset, due to the fact that images with tuna blobs also contained many other blobs that were not tuna. Subsampling and oversampling methods are available to manage unbalanced datasets [32]. For this purpose we applied a Synthetic Minority Oversampling Technique [33] to oversample the minority cases and a Spread Sample filter [34] to subsample the majority instances with the Weka software [35].

**Table 1:** Ratios between presence and absence cases for the three databases: the original database (TOTAL), a subsampled database (SMOTE) and an oversampled database (SPREAD).

	*Tuna*	*No Tuna*	*Ratio*
TOTAL	1497	21004	14.03
SMOTE	2994	21004	7.02
SPREAD	1497	10502	7.01

As a result, we constructed three training datasets: a complete dataset (TOTAL) with 22501 instances; (ii) an oversampled dataset (SMOTE) with 23998 instances; and (iii) a subsampled dataset (SPREAD) with 11999 instances, with 20 morphological characteristics in each one (Table 1).

### Model training and evaluation

A first experiment was performed to evaluate the merits of using only some of the 20 characteristics available in the dataset. We compared the classification performance of the reduced datasets containing a subset of characteristics with the performance of the dataset containing the whole set of characteristics. The subset of characteristics in the reduced datasets were selected using four attribute selection filters: ChiSquared, InfoGain, Support Vector Machine (SVM) and Stepwise [34]. The Stepwise method provided an optimum number of characteristics (13 in our case), while the rest of the attribute selection filters were applied at fixed numbers of characteristics (ranging between 3 and 19 in steps of 2). In each case, the attribute selection filter selected the most powerful combination of characteristics. On the reduced datasets, we applied the “five replications of two fold cross-validation” methodology (5x2cv). With this methodology, in each of the five replications, the available data were randomly partitioned into two equal sized datasets, a training dataset and a testing dataset, so that each data point had a chance of being validated. Using the Random Forest (RF) classification algorithm [36], a classification model was generated with each training dataset and validated on the testing dataset [37]. To compare their relative performance, the Kappa [38] and Accuracy values of the reduced datasets were compared to those of the complete dataset. A corrected resampled t-test was also performed to test the null hypothesis of whether the classification with the reduced dataset yielded the same accuracy as when using the complete dataset. This experiment was run under R [39], making calls to Weka software. BioSeqClass [34] and MASS [40] packages were used for this purpose.

Once the optimum number of characteristics was determined, five classification methods were applied to each of the three different datasets (TOTAL, SMOTE and SPREAD): RF [36], SVM [41, 42], Multilayer Perceptron (MLP) [43, 44], Iterative Dichotomiser 3 (J48 in WEKA) [45] and Instance-Based learner with fixed neighborhood (IBK) [46]. RF, MLP, IBK and J48 classifications were applied using Weka software and the SVM was applied using R software.

To evaluate the effectiveness and efficiency of classification methods we estimated the average validation indices for sensitivity, specificity, Kappa and Area Under the Curve (AUC). These validation indices are calculated using a confusion matrix which evaluates the predictive accuracy of presence-absence models on a set of test data for which the true values are known. The confusion matrix is defined by the true positive rate (TP, presence was correctly predicted by the model), the true negative rate (TN, absence was correctly predicted by the model), the false negative rate (FN, the model incorrectly predicted absence) and the false positive rate (FP, the model incorrectly predicted presence).

Sensitivity and specificity were calculated by the caret R package [47], as follows:

$$Sensitivity=\frac{TP}{TP+FN}$$ (Eq 1)

$$Specificity=\frac{TN}{FP+TN}$$ (Eq 2)

Sensitivity measures the efficiency of the algorithm in correctly classifying positive cases, and specificity measures the efficiency of the algorithm in correctly classifying negative cases.

Kappa and AUC, both are calculated by the PresenceAbsence R package [48]. Kappa is a measure of agreement between the classifications and the true classes. It's calculated as the difference between the relative observed agreements (*p*_*o*_) and the relative agreements expected by chance (*p*_*e*_) divided by the maximum possible agreement: It is known as the "chance-corrected proportion of agreement” [38] and it is calculated as follows:

$$Kappa=\frac{p_{o}-p_{e}}{1-p_{e}}$$ (Eq 3)

AUC, is a common evaluation metric for binary classification problems and represents the area under the receiver operating characteristic (ROC) curve [49]. ROC graphs are two-dimensional graphs in which the TP rate is plotted on the Y axis and the FP rate is plotted on the X axis. It ranges between 0 and 1. When the classifier is very good, the TP rate will increase quickly and the area under the curve will be close to 1. If the classifier has a random behavior, the TP rate will increase linearly with the FP rate and the area under the curve will be close to 0.5.The scale most commonly used for model evaluation implies that a model with an AUC value of 0.95 or higher is excellent; between 0.85 and 0.95 is good; between 0.75 and 0.85 is acceptable; and below 0.75 is poor [49].

The validation indices (Kappa, Sensitivity, Specificity and AUC) were computed after executing 30 runs of the classification algorithm with 10-fold cross-validation in order to avoid overfitting and to achieve stable results [50].

## Results

**Fig 4:**
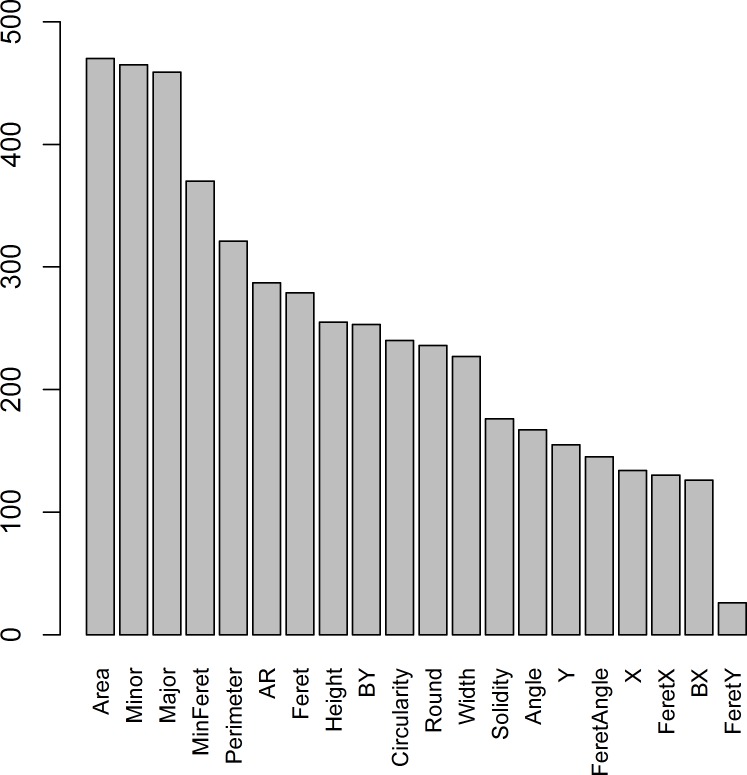
Frequency with which the blob characteristic were selected by the different attribute selection filters during the experiment to evaluate the merits of using reduced datasets.

**Fig 5:**
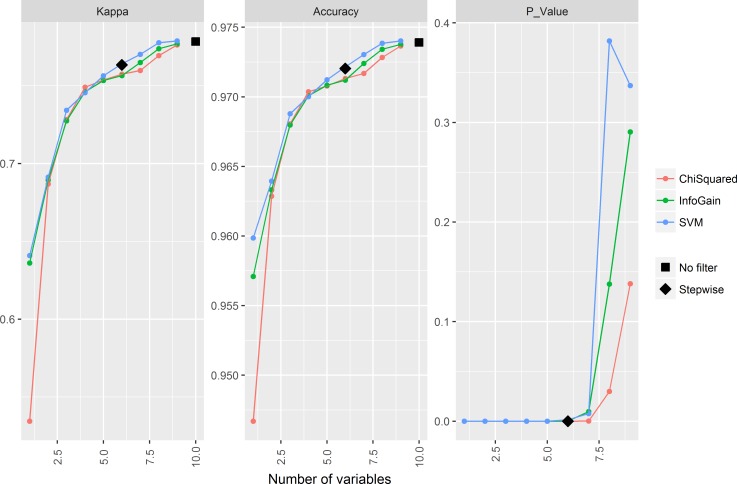
Comparison between the complete dataset and the reduced datasets. Values for *Kappa*, *Accuracy* and *P_Value* (obtained from a corrected resampled t-test) are shown.

In the first experiment, the characteristics that were most consistently selected by the different attribute selection filters were *Area*, *Major* and *Minor* (Fig 4), which are correlated, suggesting that the size of the blob is most informative about the tuna or no-tuna category. However, both Kappa and accuracy values increased as the number of characteristics increased. The trends for both Kappa and accuracy were similar: highest gains occurred for reduced number of characteristics (up until 9), but classification performance continued to gradually improve afterwards, albeit at lower rates. Overall, none of the reduced datasets (including only a subset of the characteristics) improved the performance of the complete dataset. According to statistical t-tests, a similar performance was achieved only when a high number of characteristics were included in the reduced dataset (17 or 19 characteristics, depending on the attribute selection method, Fig 5). Thus, since our main goal was to achieve the best classification performance, and computing time was not a limiting factor, we decided to use the complete dataset (with 20 characteristics) instead of a reduced dataset.

**Fig 6:**
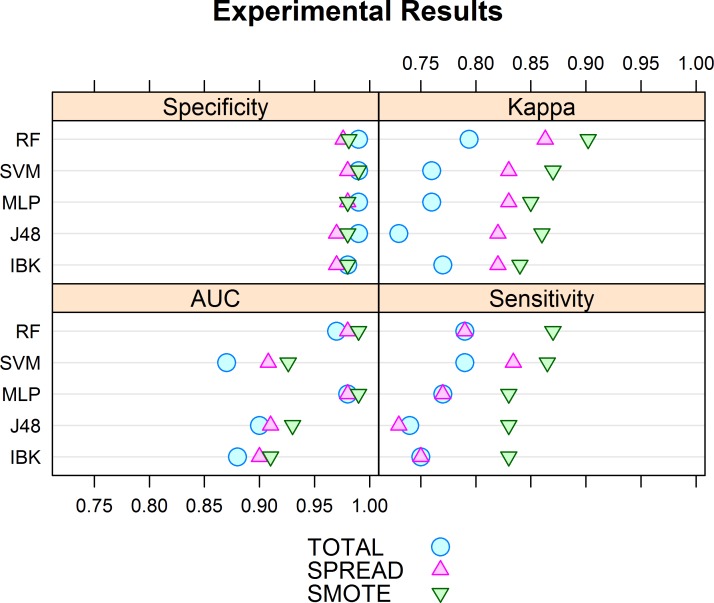
Experiment results. Specificity, sensitivity, AUC and Kappa values for the three datasets: TOTAL, a complete dataset with 22501 instances; SPREAD, an oversampled dataset with 23998 instances; and SMOTE, a subsampled dataset with 11999 instances. The Y axis represents the classification method used: Random Forest (RF), Support Vector Machine (SVM), Multilayer Perceptron (MLP), J 48 and IBK.

Regarding the bluefin tuna classification study, with the original dataset (TOTAL), acceptable results were obtained for all algorithms (Fig 6). AUC values were between 0.87 and 0.97 with a difference in performance between algorithms of around 10%, such as between SVM and MLP. Sensitivity estimates varied between 0.73 and 0.79, indicating that all algorithms classify positive (“tuna”) instances with similar efficiency. For specificity, all algorithms obtained very high results (> 0.95) with minor differences between them. Consequently, most negative (“no tuna”) instances were correctly recognized. Kappa values also ranged from 0.74 to 0.79, thus evidencing good ratios between true positives and true negatives.

For both SPREAD and SMOTE, due to the use of more balanced datasets, the results generally improved for all the indices. This was not the case for the specificity, which showed lowest variation between datasets, and were high (> 0.95) in all instances.

With the SPREAD dataset the performance of the different algorithms (as measured by AUC and sensitivity) improved with respect to the TOTAL dataset. AUC estimates ranged between 0.90 and 0.98 and were higher than with the TOTAL dataset in all instances. Sensitivity values varied from 0.82 to 0.86 and the lowest value was higher than any of the ones obtained with the TOTAL dataset. Although highest specificity and Kappa were scored by SVM, highest sensitivity and AUC values were scored by the RF.

Finally, the SMOTE dataset obtained the best general accuracy, especially in terms of sensitivity, Kappa and AUC, since scores for these three indices where higher than those obtained with the TOTAL and SPREAD datasets, in all cases. AUC, Kappa and sensitivity values varied from 0.91 to 0.99, 0.83 to 0.87, and 0.84 to 0.90 respectively. SVM showed the best specificity, but RF was the algorithm showing best AUC, sensitivity and Kappa scores.

## Discussion

A semi-automated image processing and a supervised classification validation method have been developed and applied to detect the presence/absence of bluefin tuna in sonars that are routinely used by the fishing vessels targeting this species. The results of the classification validation tests show that all algorithms have good classification efficiency. Among the three datasets used in the experiment, the TOTAL dataset obtained a good overall performance, but balanced datasets SPREAD and SMOTE subsequently improved the general performance. The RF algorithm applied to the SMOTE dataset provided the highest accuracy among the tested algorithms. Nevertheless, although different machine learning algorithms were compared, the main objective of the experiment was not to select the best algorithm. The overall good performance in classifying “tuna” and “no tuna” cases allowed us to validate the proposed methodology. The particular algorithms can be selected on a case by case basis, considering additional constraints (e.g. computing time) in particular future applications. In fact, MLP and SVM require substantially larger calculation time, which can be an additional consideration to guide selection in specific applications such as the processing of massive amounts of data (e.g. obtained from monitoring programs in the whole fleet throughout the whole fishing season), or when the speed of the analysis is critical (e.g. for near real time monitoring of resource abundance and distribution).

On one hand, the results of this work indicate that the designed methodology has an appreciable morphologic discriminatory capacity with the processed images. On the other hand, it should be taken into account that the ratio of positives and negatives in the set of images used in this experiment may not be representative of the ratio in the commercial fishing trips conducted by the baitboat fleet in the Bay of Biscay (where a higher percentage of negative cases is expected). This will have to be taken into account when the model is applied to datasets obtained e.g. during an entire fishing campaign by estimating the real presence/absence ratios and using algorithms that properly deal with uncompensated datasets. In addition, in such an extensive application of the model, the classification will have to be semi-supervised. Although a decrease in efficiency of the classification might be expected, the generalization of the model will likely increase since a larger variety of situations will be encountered [51].

In order to enhance the strengths of this methodology, several future research lines are being developed. First, following [8], a flexible Optical Character Recognition (OCR) method to extract metadata from sonar screens (sonar signal range, tilt, gains, speed, heading, as well as additional information) is being developed so that extra information can be introduced to guide classification on an image by image basis. This will also allow providing standardized tuna school sizes, since they can be specially affected by the gain settings. And second, tools for temporal tracking of schools should be used to identify the same school in a set of sequenced images. This is a necessary step in order to be able to quantify the total number of schools observed as well as to characterize their size. Additionally, these sonar observations could be paired with additional bluefin tuna presence/absence data from logbooks and/or scientific observers, as they become available, to allow a continuous improvement of the reference dataset used to train the algorithms.

A third research line will consist in combining the MRS data with scientific echosounder data [25]. The main purpose of extracting the number and size of schools from MRS screenshots is to provide an index of abundance of bluefin tuna; for instance, something of the type of a sonar mapping [52]. It is clear, though, that the data obtained from MRS images might not be as precise as those from standard acoustic-trawl surveys, based on echointegration [53] of data recorded by calibrated scientific echosounders [54]. However, currently, there are no ongoing acoustic surveys estimating the abundance of Atlantic bluefin tuna in the Bay of Biscay nor anywhere else, due to the large spatial distribution and high mobility of this species. In addition, typical single-vessel acoustic-trawl surveys have spatial-temporal limitations that could be overcome by an extensive implementation of this methodology [5]. Taking this into account, we plan to combine the extensive sonar mapping based on this methodology with the density distribution of the schools measured by a scientific echosounder. This would allow us to overcome the inherent uncertainty of the analog sonar images and also the sampling limitation of the standard, single vessel scientific echosounder acoustics. In practical terms, the low cost of the data acquisition device and the automation of the process would allow it to be applied extensively (in many vessels and through large periods of time) while carrying scientific echosounders in one or a few of the vessels. Additionally, the side-scan sonars increase the volume sampled near the surface and thus may constitute an adequate sampler of near-surface distribution species as the bluefin tuna while feeding in the Bay of Biscay. Side scan sonars have been successfully used for fishery work in other areas in the past [55, 56, 57], and allow in-season decisions on the spatial and temporal sub-allocation of the total allowable catch [7].

**Fig 7:**
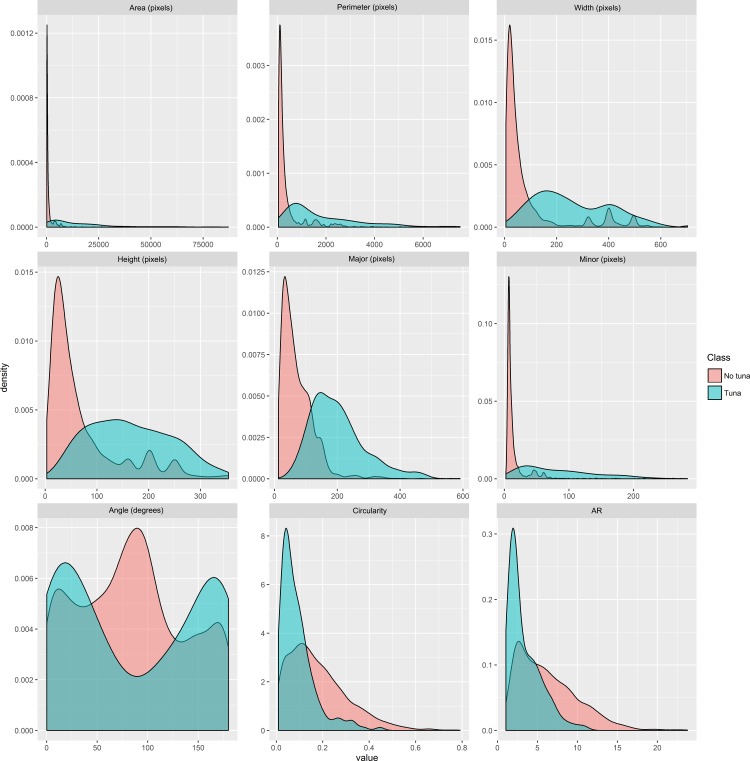
Density plots of the measured characteristics for tuna and no-tuna blobs. Only ecologically meaningful characteristics, related to size and shape of the schools, are plotted. “Angle” refers to the angle (0–180°) between the X axis of the image and the primary axis of the best fitted ellipse to the blob contour; “circularity” is proportional to the ratio between the area and the squared perimeter, with a value of 1 representing a perfect circle and a value of 0 representing an increasingly elongated shape; Aspect Ratio (AR) is the ratio between the primary and secondary axes of the fitted ellipse., The area that a single pixel represents ranges between 0.69 m2 and 0.92 m2 (depending on the gain setting of the sonar).

The morphological differences between the tuna and no-tuna blobs allow for their discrimination. In fact, tuna blobs were generally larger, more elongated and showed a more horizontal alignment. Some of these characteristics are ecologically meaningful, and the measurements obtained in the different blobs can inform e.g. about the size and shape of the bluefin tuna schools aggregated in the Bay of Biscay during the summer feeding season (Fig 7). Bauer et al [58] classified the size of tuna schools based on the surface disturbance observed by airplanes. Similarly, the measurements of the area of the blobs classified as “tuna” could be used to provide estimates of the size of the different schools in the future.

The Standing Committee on Research and Statistics of ICCAT has recurrently highlighted the need for developing fishery independent indices of abundance, given the problems associated with existing CPUEs and their inability to accurately track biomass changes, especially in recent years after the implementation of the recovery plan [14]. Our study can be considered a first milestone towards getting more accurate indices of abundance for juvenile bluefin tuna in the Bay of Biscay, and this can be pursued in two ways: (i) On one hand, the automated procedure presented in our study could be applied to MRS images recorded onboard commercial fishing vessels during their commercial operations. The bluefin tuna detections per unit of effort (DPUE, in number of schools per time unit) could be standardized, just in a similar way to the CPUE observations of the commercial fleet [22], currently used in the bluefin tuna assessment model. The signal of inter annual variability in bluefin tuna abundance can be isolated by removing the variability in DPUE due to other variables like month, area, or skipper skill, and this time series of standardized DPUE could be used as an index of relative abundance to tune the stock assessment models. Compared to the standardized CPUE that is currently used, the standardized DPUE index would have the advantage that the detections by the sonar, unlike the catch, would be independent from factors affecting the bluefin catchability by baitboats, such as the availability of tuna forage in the environment. It would, therefore, in principle better reflect the real abundance of bluefin tuna in the Bay of Biscay, compared to the standardized CPUE that is based on what the baitboat fleet was able to finally catch. However, the variability of factors affecting detection of bluefin tuna by the sonars would need to be considered in the DPUE standardization process. Since vessels could use different sonar settings at different times, it is important to standardize DPUE observations to standard sonar range, tilt, and gain values.

(ii) On the other hand, transect based systematic surveys covering the Bay of Biscay onboard commercial baitboats equipped with MRS could be designed and conducted yearly to quantitatively estimate the bluefin tuna school density (in number of schools per area unit). Such a time series could be used as a relative index of abundance to tune stock assessment models. Given the relatively high mobility of tunas (compared to small pelagics or demersal resources), ideally the systematic surveys would involve several commercial boats equipped with MRS so that the whole area of distribution can be searched in few days. This is advantageous compared to when a single boat prospects all the area (which is often the case when scientific acoustic equipment is used to estimate total biomass), because the probabilities of immigration, emigration and double counting schools are diminished. Bluefin tuna concentrate in a relatively small area while feeding during summer in the Bay of Biscay (Fig 1), which provides a unique opportunity to conduct systematic abundance surveys on this widely distributed species [30].

The relatively non-expensive methodology presented in this study can also be adjusted to other tuna and non-tuna pelagic fisheries by adapting the analyses to the specific type of sonar, output signal and display (see also [8]). This provides an interesting alternative to standard acoustic-trawl surveys, especially when targeting species of high mobility and/or near surface distribution. It thus provides an opportunity to use commercial fishing vessels as observatories of the pelagic ecosystem, and commercial sonars as tools to track changes in abundance of commercial species [6, 8].

## Supporting information

S1 Dataset Complete training dataset of tuna and no-tuna blobs. “Blob_ID” is a unique blob identifier (as a concatenation of survey, year, time and blob number); “Area” and “Perimeter” of the blob are in number of pixels; “BX” and “BY” refer to the upper left corner coordinates of the smallest rectangle housing the blob; width and height refer to the dimensions (in pixels) of such rectangle; “X”, “Y”, “Major”, “Minor” and “Angle” refer to the coordinates of the centroid, the size of the principal and secondary axes, as well as the angle (with respect to the horizontal axis) of the best fitting ellipse; “Circularity” is proportional to the ratio between the area and the squared perimeter, with a value of 1 representing a perfect circle and a value of 0 representing an increasingly elongated shape; Feret’s diameter, or “Feret” is the longest distance between any two points along the selection boundary, also known as maximum caliper; “FeretAngle” is the angle (0–180 degrees) of the Feret’s diameter and “MinFeret” is the minimum caliper diameter; “FeretX” and “FeretY” refer to the starting coordinates of the Feret diameter; “Aspect Ratio” (AR) is the ratio between the primary and secondary axes of the fitted ellipse; “Roundness” is the inverse of AR; “Solidity” is the ratio between the area and the convex area of the blob; and “Class” refers to the “tuna” or “no-tuna” category.
(ZIP)
